# *In silico* analysis of insect-associated bacterial phytases reveals optimal biochemical properties and function in poultry gut condition

**DOI:** 10.1093/bioadv/vbaf256

**Published:** 2025-10-15

**Authors:** Olyad Erba Urgessa, Ketema Tafess Tulu, Mesfin Tafesse Gemeda, Hunduma Dinka

**Affiliations:** School of Biological Sciences and Biotechnology, College of Natural and Computational Sciences, Haramaya University, Oromia, P.O.BOX 138, Dire Dawa, Ethiopia; Department of Applied Biology, School of Applied Natural Science, Adama Science and Technology University, Oromia, P.O.BOX 1888, Adama, Ethiopia; Department of Applied Biology, School of Applied Natural Science, Adama Science and Technology University, Oromia, P.O.BOX 1888, Adama, Ethiopia; Institute of Pharmaceutical Science, Adama Science and Technology University, Oromia, P.O.BOX 1888, Adama, Ethiopia; Department of Biotechnology, College of Natural and Applied Sciences, Addis Ababa Science and Technology, Addis Ababa, P.O.BOX 16417, Addis Ababa, Ethiopia; Department of Applied Biology, School of Applied Natural Science, Adama Science and Technology University, Oromia, P.O.BOX 1888, Adama, Ethiopia

## Abstract

**Motivation:**

Insect guts may harbor phytase-producing bacteria applicable in poultry nutrition, but only *Serratia* sp. TN49 and its histidine acid phytase (AEQ29498.1) have been studied for this purpose. Therefore, AEQ29498.1 was used as a query to conduct a homology search for insect-associated bacterial phytases, followed by prediction of their structure and function. This *in silico* analysis of phytase may lead to the isolation of native phytase-producing bacteria from insect guts, potentially facilitating the production of desirable phytases for use in feed additives.

**Results:**

Twenty-six phytases from bacteria associated with the guts of black soldier fly larvae, fruit flies, and honey bees were identified. The mature chains of these phytases, except for the 4-phytase of *Bartocella apis* PEB0150, were predicted to carry a positive charge under the acidic conditions of the poultry upper gastrointestinal tract. They are stable (instability indices <40) and belong to histidine acid phosphatase family, which has been proven to be an effective poultry feed additive. The three-dimensional structure of the mature histidine-type phosphatase of *Tatumella* sp. JGM130 demonstrated the best quality and was found to be a homo-tetrameric protein. Molecular docking confirmed phytate binding at the catalytic motif of the histidine acid phosphatase family, RHGVRPP/AP/Q and HD.

## 1 Introduction

Insects are regarded as the most successful group of animals on Earth, a success driven by several key factors, including their diverse feeding strategies that enable them to thrive in numerous ecological niches (Lu *et al.* 2013). The insect gut is a dynamic and complex ecosystem that harbors a diverse array of microorganisms, including bacteria, fungi, archaea, and viruses (Huerta-García and Álvarez-Cervantes 2024). Among these, bacteria are the most abundant and play a critical role in shaping the physiology, ecology, and evolution of their insect hosts (Lu *et al.* 2013). The composition of gut-associated bacteria varies widely across insect species, influenced by factors such as diet, habitat, developmental stage, and evolutionary history (Huerta-García and Álvarez-Cervantes 2024).

Insects that feed on plants may harbor phytase-producing bacteria capable of breaking down plant phytate (Zhang *et al.* 2011). Phytate and phytase play critical and interconnected roles in poultry nutrition, particularly with respect to phosphorus availability, nutrient absorption, and environmental sustainability. Phytate (or phytic acid) is the primary storage form of phosphorus in many plant seeds and grains, especially cereal grains, oilseeds, and legumes (Kumar *et al.* 2019). These ingredients are common sources of energy and protein in poultry diets. However, phytate poses several challenges for poultry nutrition. Poultry, like other monogastric animals, lack sufficient endogenous phytase; consequently, the phosphorus bound within phytate is poorly available to the bird (Dersjant-Li *et al.* 2015). Moreover, phytate has anti-nutritional effects: it chelates (binds) essential minerals such as calcium, zinc, and iron, and it can reduce the digestibility of proteins and starch (Woyengo and Nyachoti 2013).

Phytase plays a vital role in poultry nutrition by hydrolyzing phytate and releasing bound phosphorus and other nutrients (Dersjant-Li *et al.* 2015, Urgessa *et al.* 2024). Supplementing poultry diets with phytase enhances phosphorus digestibility and absorption, reduces the need for inorganic phosphate supplements (e.g. dicalcium phosphate), and lowers feed costs. Additionally, phytase liberates minerals such as calcium, zinc, magnesium, and iron that are bound by phytate, and it improves the digestibility of amino acids and energy (Dersjant-Li *et al.* 2015).

Beyond its nutritional benefits, phytase offers significant environmental advantages by reducing phosphorus excretion into the environment, thereby minimizing pollution such as eutrophication. Economically, phytase supplementation lowers feed costs and improves growth performance, making it a highly cost-effective feed additive (Woyengo and Nyachoti 2013).

**Figure 1.**
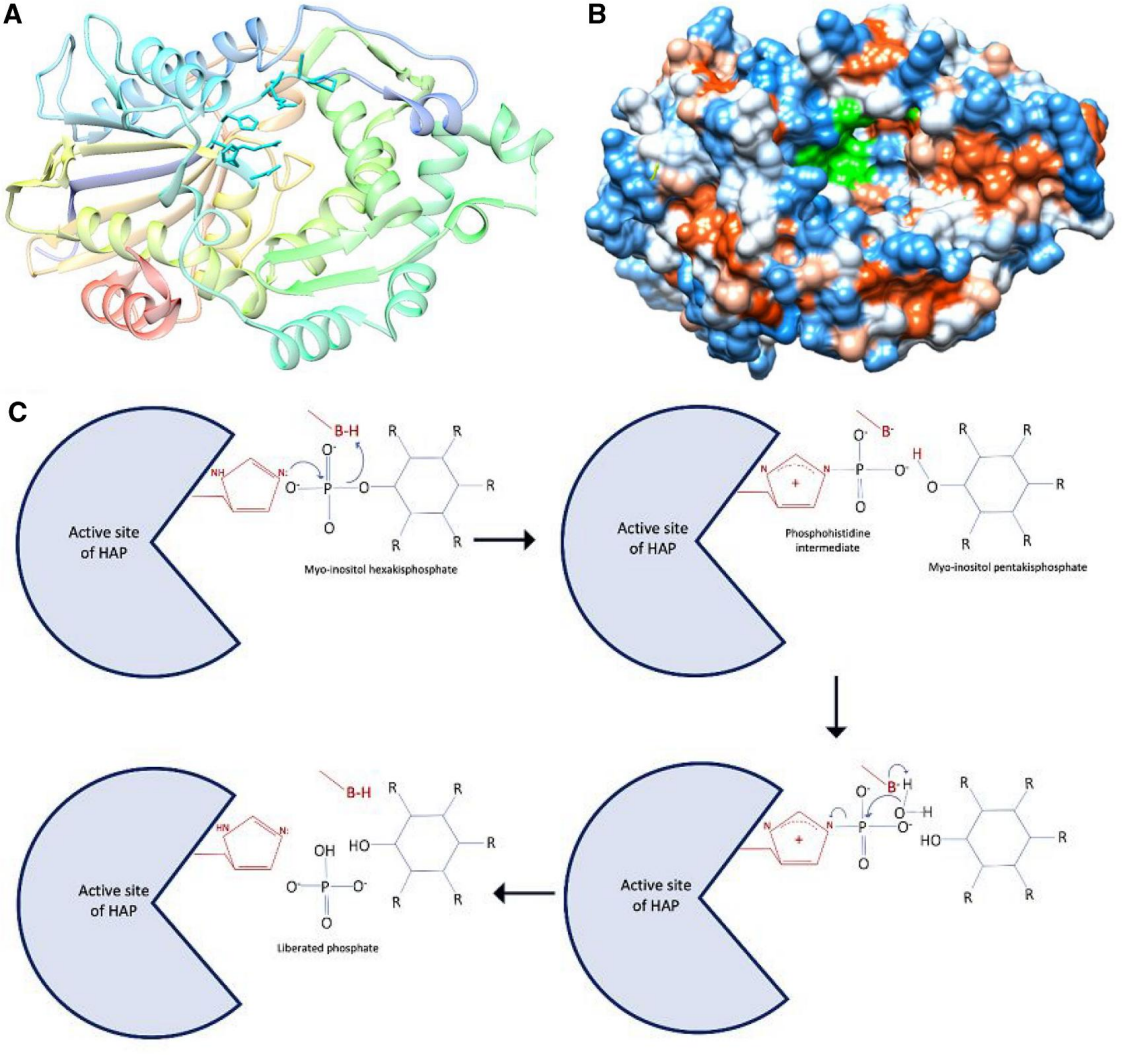
The structure of histidine acid phytase and the reaction it catalyzes. (A) Ribbon representation of histidine acid phytase with stick cyan shows active site. (B) Hydrophobicity surface of the phytases with green hollow shows active site. (C) Catalytic mechanism of histidine acid phosphatases showing the two steps. The histidine acid phytase was retrieved from Protein DataBank (PDB: 1DKL), and visualized using UCSF Chimera (Pettersen *et al.* 2004), and the catalytic mechanism was taken from Nezhad *et al.* (2020).

There are four types of phytases based on their protein domains: Histidine Acid Phytases (HAPs), Beta-Propeller Phytases (BPPs), Purple Acid Phosphatase (PAP) Phytases, and Protein Tyrosine Phosphatase-like (PTP-like) Phytases (Mullaney and Ullah 2003, Chen *et al.* 2015, Kumar *et al.* 2017, Scott *et al.* 2024). HAPs are characterized by a conserved histidine acid phosphatase domain and typically have an active site containing a histidine residue involved in catalysis. BPPs contain a beta-propeller fold domain, featuring a distinctive structure formed by repeating beta-sheet blades arranged in a propeller shape. PAPs possess a metallophosphatase domain that includes a binuclear metal center, usually consisting of iron and other metals. Lastly, PTP-like phytases have a domain similar to protein tyrosine phosphatases (Chen *et al.* 2015). The structure and reaction of HAPs are shown in Fig. 1. The histidine residue of the conserved RHGxRxP motif acts as a nucleophile and forms phosphohistidine intermediate. This intermediate is hydrolyzed after a water molecule attacks the phospho group of the phosphohistidine, generating a free phosphate (Nezhad *et al.* 2020).

The whole genome of insect gut-associated bacteria can be mined for phytase genes, addressing the gap in lab-based studies (Segers *et al.* 2017, McMullen *et al.* 2021). *In silico* protein analysis further aids in understanding the structural and functional properties of these enzymes. Molecular modeling, sequence alignment, and structural bioinformatics enable researchers to examine key enzyme features—such as active site configurations, stability, and interaction dynamics—without requiring physical isolation (Niño-Gómez *et al.* 2017, Pramanik *et al.* 2018b). Notably, *in silico* approaches have greatly advanced the study and refinement of phytases for animal feed applications (Nezhad *et al.* 2020). Accordingly, this research aimed to perform a homology search for phytase enzymes in insect-associated bacteria, followed by the prediction of their biochemical, structural, and functional properties in relation to the conditions of the upper gastrointestinal tract of poultry.

## 2 Methods

### 2.1 Similarity searching and phylogenetic analysis

First, the HAP sequence (GenBank accession no.: JF720041) was retrieved from the NCBI nucleotide database (https://www.ncbi.nlm.nih.gov/nuccore/). The coding sequence (CDS) (accession no.: AEQ29498.1) was selected, and the “Run BLAST” option under “Analyze this sequence” was clicked. On the BLAST homepage (https://blast.ncbi.nlm.nih.gov/Blast.cgi), a BLASTp analysis was performed, excluding nonredundant RefSeq protein sequences (WP) to get the source of each sequence. The accession numbers of each sequence were reviewed, and the sources of the bacterial isolates were identified from the Feature, Bioproject, or Biosample lines in the GenBank file. Sequences from insect-associated bacteria were then selected and downloaded in both FASTA complete sequence and description table formats. Finally, all retrieved sequences were cross-checked in the Universal Protein Database (https://www.uniprot.org) to verify their entry status, determine if they were obsolete, and identify the presence of predicted signal peptides.

The percent similarity index among the sequences was calculated to quantify their degree of relatedness. This was done using the Multiple Sequence Comparison by Log-Expectation (MUSCLE) algorithm, accessed through the European Bioinformatics Institute (EBI) web service (https://www.ebi.ac.uk/jdispatcher) (Madeira *et al.* 2024). A phylogenetic tree was constructed using MEGA 11 software (Tamura *et al.* 2021) for further analysis of sequence relatedness. To begin, a new protein sequence alignment was created by importing the sequence file, selecting all sequences, and aligning them using the inbuilt MUSCLE algorithm. The resulting alignment was saved as a MEGA session file. Next, the saved multiple sequence alignment was reopened using the Analysis Tab in MEGA 11, and a phylogenetic tree was generated using the Neighbor-Joining (NJ) statistical method. For the analysis, the JTT (Jones-Taylor-Thornton) substitution model was chosen as the evolutionary model due to its suitability for protein sequence data. To evaluate the reliability and robustness of the tree topology, a bootstrap analysis was conducted with 1000 replicates, providing statistical support for the branching patterns. The rate variation among sites was modeled with a gamma distribution (shape parameter = 1), and all ambiguous positions were removed for each sequence pair (pairwise deletion option).

### 2.2 Signal peptide prediction

The presence of signal peptides in the phytase sequences was predicted using two computational tools: SignalP 6.0 (https://services.healthtech.dtu.dk/services/SignalP-6.0/) (Teufel *et al.* 2022) and Phobius (https://phobius.sbc.su.se/) (Krogh *et al.* 2007). For the SignalP 6.0 prediction, a local file containing the sequences was uploaded to the web server and submitted for prediction using the default settings. For the Phobius prediction, the sequence file was also uploaded to the server, and the output format was toggled to “long with graphics.” The prediction was then run under the “normal prediction” mode.

### 2.3 Biochemical property prediction

The biochemical (physicochemical) properties of the phytase sequences were determined using the ExPASy ProtParam tool (https://www.expasy.org/protparam) (Gasteiger et al. 2005). For the analysis, each phytase sequence was input individually into the tool, and the software computed parameters including molecular weight (MW), theoretical isoelectric point (pI), amino acid composition, atomic composition, extinction coefficient, estimated half-life, instability index (II), aliphatic index (AI), and grand average of hydropathicity (GRAVY).

### 2.4 Tertiary and quaternary structure prediction

Homology-based three-dimensional phytase modeling was conducted for the HAP of Serratia sp. TN49 (AEQ29498.1), histidine-type phosphatase (HTP) of *Klebsiella pneumoniae* (UOB87035.1), HTP of Tatumella sp. JGM130 (MBS0894709.1), and HTP of *Bartonella apihabitans* W8097 (MBI0020902.1) using the Swiss-Model Workspace (https://swissmodel.expasy.org) in automated mode (Waterhouse *et al.* 2018). The sequences, either a single mature chain or full-length phytase, were input and queried against the Swiss-Model Template Library (SMTL). The suitable templates were identified and utilized for model building. The final phytase models were chosen based on the highest Global Model Quality Estimate (GMQE), Quality Model Energy Analysis based on Distance Constraint (QMEANDisCo) Global, Quality Model Energy Analysis (QMEAN), and Quaternary Structure Quality Estimate (QSQE). The best quality model was submitted to the ModelArchive database and is available at https://www.modelarchive.org/doi/10.5452/ma-hvsye.

### 2.5 Families and functional property prediction

The superfamily and functional motifs of the phytase sequence were identified using InterPro (https://www.ebi.ac.uk/interpro) (Blum *et al.* 2025). Under the “Search by Sequence” tab, the phytase sequence was input and analyzed to determine its family, domain, and motifs.

### 2.6 Substrate and phytase molecular docking

Molecular docking was performed using the SwissDock server (http://www.swissdock.ch) with the Attracting Cavities 2.0 (AC) algorithm (Röhrig et al. 2023, Bugnon et al. 2024). The Simplified Molecular Input Line Entry System (SMILES) notations of the substrates (ligands) such as phytic acid (CID: 890) and 4-nitrophenyl phosphate (CID: 378) were obtained from the PubChem database (https://pubchem.ncbi.nlm.nih.gov/). These SMILES notations were individually copied and pasted into the ligand box, followed by activation of the red bar for preparation.

For the receptor part, Chain A of the homotetrameric and monomeric phytases were uploaded and processed. The Swiss models were opened in UCSF Chimera, and chains B, C, and D were deleted, leaving Chain A, which was then saved as a separate PDB file. Next, the X, Y, and Z coordinate values of the last atoms of each residue in the catalytic domain were averaged, and these values were entered into the respective box centers in SwissDock. Default docking parameters were applied.

The docking results were downloaded as a zip file and extracted. Using UCSF Chimera, dock4 results from the folder was opened, followed by the receptor.pdb file from the extracted file folder. Out of 49 default poses, the one that best fit the catalytic motif was selected and saved separately. This result was visualized again in UCSF Chimera, with the active sites shown in stick representation. The ligand was selected, and its contacts with the phytase were designated and saved using UCSF Chimera. The saved files were then imported into Microsoft Excel to counting the interacting residues. Final docking results were saved as images using UCSF Chimera for presentation.

## 3 Results

### 3.1 Homology and phylogenetic analysis for insect-associated bacterial phytase

Using BLASTp homology research with a HAP (AEQ29498.1) query, a total of 99 sequence hits were identified, of which 27 sequences originated from insect-associated bacteria (26 from the gut and 1 from the mouth). The *E*-values ranged from 5 × 10^−154^ to 1 × 10^−128^, and the percent identity ranged from 45.02% to 52.52%, suggesting that these 27 sequences are homologous phytases. A percent identity ≥30% and an *E-*value ≤1 × 10^−6^ are generally considered significant indicators of homology (Pearson 2013). The HAP was isolated from *Serratia* sp. TN49, a bacterium isolated from the midgut of *Batocera horsfieldi* larvae (Zhang *et al.* 2011). The other phytase sequences were translated from genes in bacteria associated with the gut of fruit fly (*Drosophila melanogaster*), black soldier fly larvae (*Hermetia illucens*), honey bees (*Apis mellifera*), and the mouth of honey bees (Table 1).

**Table 1.** Homologous phytase enzyme from insect-associated bacteria.

Types of phytases	Bacterial isolate	E value	% Ident.	AA len	NCBI acc. no.	UniProt acc. no.
Histidine acid phytases	*Serratia* sp. TN49^a^	0	100	429	AEQ29498.1	G4WZU3
Histidine-type phosphatase	*Klebsiella pneumoniae* ^b^	5 × 10^−154^	52.52	430	UOB87035.1	UPI001FAFD5A8
Histidine-type phosphatase	*Tatumella* sp. JGM130^c^	1 × 10^−142^	51.91	434	MBS0894709.1	UPI001BB070F5
Histidine-type phosphatase	*T.* sp. JGM82^c^	2 × 10^−142^	51.91	434	MBS0878474.1	UPI001BB070F5
Histidine-type phosphatase	*T*. sp. JGM16^c^	6 × 10^−141^	51.67	434	MBS0857106.1	UPI001BAF83B7
Histidine-type phosphatase	*B.* sp. M0177^d^	9 × 10^−138^	47.09	462	MBI0004047.1	UPI0018DBF5B3
Histidine-type phosphatase	*B. apihabitans* W8097^d^	2 × 10^−133^	49.38	505	MBI0020902.1	No entry
4-Phytase/acid phosphatase	*B. apis* PEB0150^d^	2 × 10^−133^	46.12	462	OLY45882.1	UPI0009657BF1
Histidine-type phosphatase	*B.* sp. W8167^d^	2 × 10^−132^	49.14	496	MBI0168642.1	No entry
Histidine-type phosphatase	*B. apis* MRS1-bin.1^d^	2 × 10^−132^	45.74	462	MCT6861532.1	No entry
Histidine-type phosphatase	*B.* sp. W8122^d^	6 × 10^−132^	49.01	496	MBI0002091.1	UPI0018DE1DCC
Histidine-type phosphatase	*B. choladocola* W8125^d^	8 × 10^−132^	48.15	489	MBI0141245.1	No entry
Histidine-type phosphatase	*Rosenbergiella nectarea* subsp. *apis* B1A^e^	1 × 10^−131^	50.12	427	MBT0729351.1	No entry
Histidine-type phosphatase	*B*. *apis* GUT-bin.12^d^	1 × 10^−131^	45.74	462	MCT6887820.1	No entry
4-Phytase/acid phosphatase	*B. apis* PEB0149^d^	2 × 10^−131^	45.93	462	OLY43825.1	A0A1R0FA20
Histidine-type phosphatase	*B. apis* MRS2-bin.4^d^	3 × 10^−131^	45.74	462	MCT6825384.1	No entry
4-Phytase/acid phosphatase	*B. apis* PEB0122^d^	3 × 10^−131^	45.02	462	OLY47475.1	UPI000962EFB3
Histidine-type phosphatase	*B.* sp. M0283^d^	7 × 10^−131^	48.15	496	MBI0162225.1	No entry
Histidine-type phosphatase	*B. choladocola* B10834H15^d^	1 × 10^−130^	48.40	496	MBH9976047.1	No entry
Histidine-type phosphatase	*B. apis* W8099^d^	2 × 10^−130^	49.37	496	MBI0177545.1	No entry
4-Phytase/acid phosphatase	*B. apihabitans* BBC0244^d^	6 × 10^−130^	48.15	505	AQT45424.1	UPI00098F5FB7
Histidine-type phosphatase	*B. apihabitans* M0187^d^	7 × 10^−130^	49.75	496	MBI0025375.1	No entry
4-Phytase/acid phosphatase	*B. apihabitans* BBC0178^d^	8 × 10^−130^	47.46	523	AQT43185.1	A0A1U9MD12
Histidine-type phosphatase	*B. apihabitans* M0280^d^	2 × 10^–129^	48.87	496	MBI0166446.1	No entry
Histidine-type phosphatase	*B. apis* B10834G6^d^	2 × 10^–129^	48.15	496	MBH9982862.1	No entry
4-Phytase/acid phosphatase	*B. choladocola* BBC0122^d^	3 × 10^–129^	50.67	489	AQT47836.1	A0A1U9MJ04
Histidine-type phosphatase	*B.* sp. P0291^d^	1 × 10^–128^	48.01	505	MBH9993889.1	UPI0018DB35DE

a Bacterial isolate from gut of *B. horsfieldi larva.*

b Bacterial isolate from gut of *H. illucens larva.*

c Bacterial isolate from gut of *D. melanogaster.*

d Bacterial isolate from gut of *A. mellifera.*

e Bacterial isolate from mouth of honeybees.

The protein existence of AEQ29498.1 has been documented in UniProtKB (accession no.: G4WZU3) as inferred from homology (PE = 3), alongside phytases from *B. apis* PEB0149 (accession no.: A0A1R0FA20), *B. apihabitans* BBC0178 (accession no.: A0A1U9MD12), and *B. choladocola* BBC0122 (accession no.: A0A1U9MJ04) (Table 1). There is experimental evidence supporting the existence of G4WZU3. At the gene level, its sequence was amplified, cloned, and verified through sequencing. At the protein level, the recombinant AEQ29498.1 was expressed in *Escherichia coli*, purified using Nickel Nitrilotriacetic Acid (Ni–NTA) agarose affinity chromatography, and its apparent molecular mass was determined by SDS–PAGE. The protein’s identity was further confirmed by Liquid Chromatography–Electrospray Ionization–Mass Spectrometry (LC–ESI–MS/MS). Finally, biochemical analyses demonstrated the activity of G4WZU3, confirming its effectiveness for application in poultry feed (Zhang *et al.* 2011).

**Figure 2.**
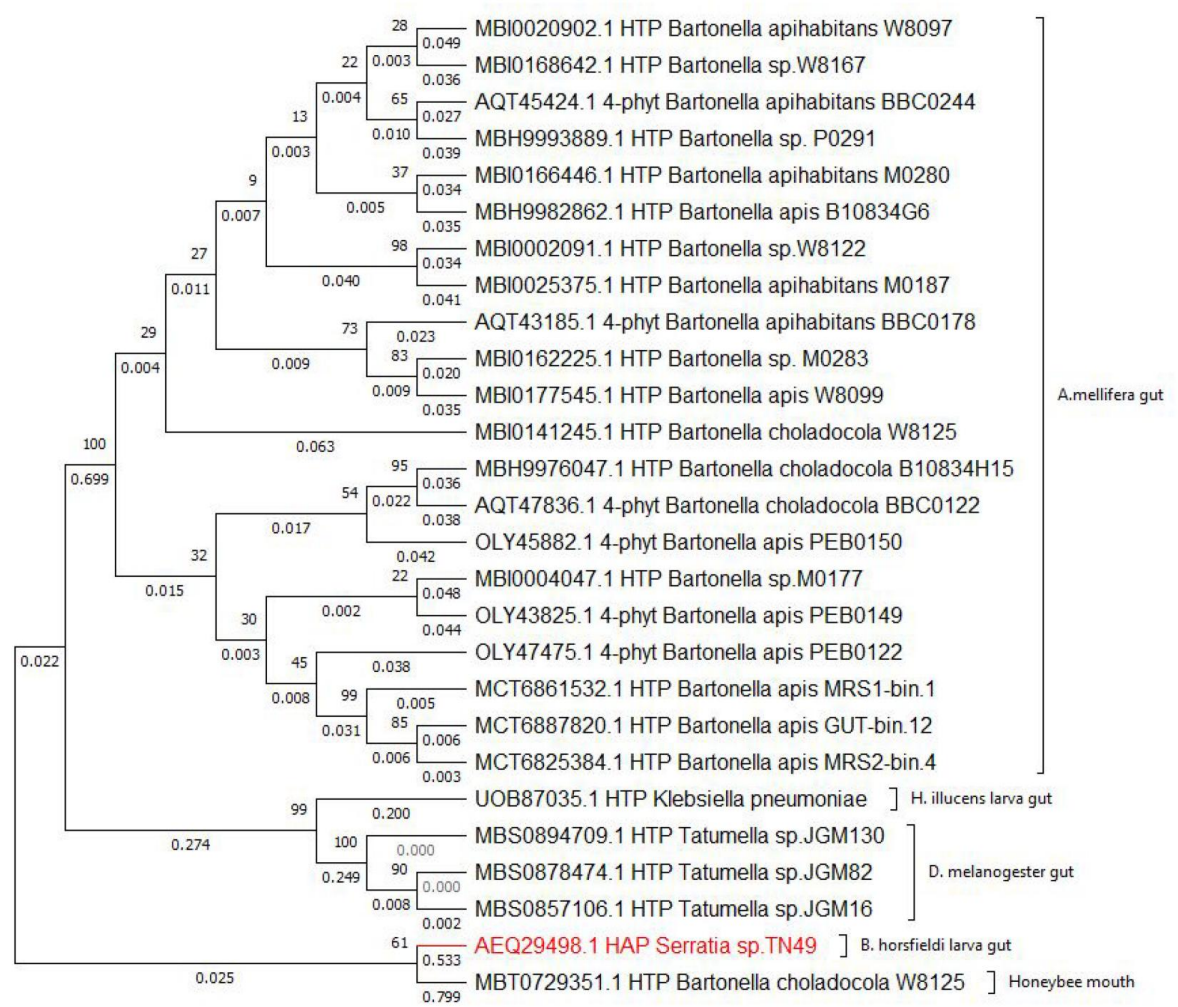
An unrooted phylogenetic tree of phytase from insect-associated bacteria. The percentage of replicate trees in which the associated taxa clustered together in the bootstrap test (1000 replicates) are shown next to the branches, and the branch lengths are shown below the branch.

The percent identity matrix generated using the MUSCLE revealed a range of identities, from the lowest value of 45.45% between the HTP of *Bartonella* sp. W8122 (MBI0002091.1) and HTP *Rosenbergiella nectarea* subsp. *apis* B1A (MBT0729351.1) to the highest value of 99.77% between HTP *Tatumella* sp. JGM16 (MBS0857106.1) and HTP of *Tatumella* sp. JGM82 (MBS0878474.1) (Supplementary Table 1, available as supplementary data at *Bioinformatics Advances* online). Unlike previous studies that explored the evolutionary relationships among bacterial species or strains based on their phytase sequences (Pramanik *et al.* 2018a, 2018b), an unrooted phylogenetic tree, which shows only the relationships and clustering patterns (Kinene *et al.* 2016) among the 27 homologous phytases, was constructed in this study (Fig. 2). AEQ29498.1 was grouped with MBT0729351.1. UOB87035.1, MBS0894709.1, MBS0857106.1, and MBS0878474.1 were clustered together with strong branch pattern support (Bootstrap values = 99, 100, and 90).

### 3.2 Signal peptide prediction for insect-associated bacterial phytases

Signal peptide analysis is crucial for predicting protein localization, function, and behavior, as these sequences direct proteins to their correct cellular destinations—such as secretion, membrane insertion, or organelle targeting. In biotechnology, optimizing signal peptides can improve recombinant protein yield and stability. They also help infer whether a protein acts extracellularly or intracellularly (Owji *et al.* 2018). In this study, signal peptides were predicted using SignalP 6.0 and Phobius, yielding consistent results for 11 sequences (Supplementary Table 2, available as supplementary data at *Bioinformatics Advances* online). All the predicted signal peptides were of the Sec/SPI type (Teufel *et al.* 2022). Phobius predicted signal peptides spanning residues 1 to 37 for most phytases, while SignalP 6.0 predicted them from residues 1 to 43. The six-residue difference was mostly identified as KPEAIA. A 25-residue signal peptide was predicted for AEQ29498.1, aligning with annotations in the UniProtKB database. However, Zhang *et al.* (2011) previously reported a 31-residue signal peptide for AEQ29498.1, with the discrepancy likely attributed to advancements in signal prediction tools (Teufel *et al.* 2022). Interestingly, the signal peptide prediction analysis revealed identical signal peptides for the three HTPs (MBS0894709.1, MBS0878474.1, and MBS0857106.1) belonging to *Tatumella* species.

### 3.3 Biochemical properties of insect-associated bacterial phytases

Predicting a protein’s biochemical properties such as molecular weight (MW), isoelectric point (pI), absorbance (Ab), instability index (II), aliphatic index (AI), and GRAVY, is crucial (Pramanik et al. 2018b). MW is essential for identifying and verifying proteins through methods like SDS-PAGE, mass spectrometry, or Western blot, and it helps determine the amount of protein of needed in experiments. It is also important for protein purification, size-based separation, and estimating diffusion rates (Tomii 2019). The predicted MWs of the mature phytase chains ranged from 44.14 kDa for AEQ29498.1 to 51.33 kDa for HTP of *Bartonella* sp. P0291 (MBH9993889.1) (Table 2), with their corresponding MWs of the full-length phytases being 46.638 kDa and 56.118 kDa, respectively (Supplementary Table 3, available as supplementary data at *Bioinformatics Advances* online). A similar MW for AEQ29498.1 has been documented in the UniProtKB database, although Zhang *et al.* (2011) reported a slightly higher MW of 48 kDa for the same protein.

**Table 2.** Biochemical property of mature phytase chains of insect-associated bacteria.^a^

No	NCBI accession	MW	pI	Ab		II	AI^b^	GRAVY
				S-S	SH	<40		
1	AEQ29498.1	44.14	8.96	1.218	1.210	38.31	79.18	−0.324
2	UOB87035.1	44.44	6.41	1.300	1.292	37.97	77.66	−0.404
3	MBS0894709.1	44.90	8.20	1.320	1.312	37.16	83.80	−0.306
4	MBS0878474.1	44.95	8.46	1.319	1.310	36.39	82.85	−0.317
5	MBS0857106.1	44.89	8.16	1.320	1.312	36.76	82.85	−0.300
6	MBI0020902.1	50.84	6.31	1.116	1.109	29.56	77.94	−0.510
7	OLY45882.1	46.32	5.47	1.193	1.185	33.53	81.72	−0.382
8	MBT0729351.1	44.15	6.96	1.319	1.311	42.94	83.79	−0.150
9	OLY43825.1	46.01	5.62	1.201	1.193	35.63	81.74	−0.356
10	MBI0177545.1	50.05	5.71	1.134	1.127	36.22	77.33	−0.484
11	MBI0025375.1	49.93	5.72	1.137	1.129	33.85	79.27	−0.495
12	MBH9993889.1	51.33	6.07	1.213	1.206	34.93	74.59	−0.598

a Ab is predicted light absorbance at 280 nm in water by 1 g/L of matured phytase assuming all pairs of Cys residues form cystine (S-S), or are reduced (SH).

b Abbreviations: AI = aliphatic index, GRAVY = grand average of hydropathicity, II = instability index, MW = molecular weight in kDa, pI = isoelectric point in pH.

The isoelectric point (pI) is the pH at which a protein has no net charge, influencing its solubility and interactions, and is used in purification techniques such as isoelectric focusing and ion-exchange chromatography (Tomii 2019). The predicted pI of the mature AEQ29498.1 chain was pH 8.96, representing the highest value, whereas the predicted pI of the mature 4-phytase chain (OLY45882.1) from the *B. apis* PEB0150 was pH 5.47, the lowest among the studied sequences. The corresponding pI values for their full-length phytase sequences were pH 9.05 and 7.56, respectively (Supplementary Table 3, available as supplementary data at *Bioinformatics Advances* online). All mature phytase chains, except OLY45882.1, are expected to be positively charged at pH ≤ 5.5 based on the principle that a protein carries a net positive charge when its pI is greater than the environment pH, and conversely, it is negatively charged if its pI is lower than the environmental pH (Alcântara Pessôa Filho *et al.* 2011).

Absorbance at 280 nm quantifies protein concentration based on aromatic residues, enabling quick, nondestructive measurement and monitoring of purification or degradation (Tomii 2019). Oxidized mature phytases have higher absorbance than reduced mature phytase (Table 2). Phytase retrieved from the genomes of *Tatumella* species showed higher absorbance values.

The instability index (II) predicts protein stability *in vivo* from its sequence, guiding protein engineering and storage decisions, with values above 40 indicating instability. All mature phytase chains showed II < 40, except MBT0729351.1. This indicates that these phytases are stable *in vivo* and have dipeptide compositions that contribute to stability (Guruprasad *et al.* 1990). Notably, AEQ29498.1, UOB87035.1, MBS0878474.1, and MBI0020902.1 were identified as stable phytases, each derived from bacteria associated with different insects. However, the stability of these phytases under in vitro conditions may not be conclusive, as protein stability depends not only on the intrinsic properties of the protein but also on the conditions of its surrounding environment (Gamage *et al.* 2019).

The aliphatic index (AI) relates to thermostability by measuring the volume of aliphatic side chain, aiding in engineering proteins for high-temperature environments and predicting stability in thermophiles (Tomii 2019). The AI of the mature phytase chains ranged from 77.33 to 83.80. The mature MBS0894709.1 chain may have the highest thermostability, a higher AI indicates a greater proportion of aliphatic amino acids in the protein, correlates with increased thermal stability (Ikai 1980).

The GRAVY score reflects protein hydrophobicity or hydrophilicity, helping predict solubility, membrane association, and informing buffer design during purification (Wang *et al.* 2021). All mature phytase chains were likely globular and hydrophilic proteins (GRAVY < 0). The mature MBH9993889.1 chain was the most hydrophilic (GRAVY = −0.598), while the mature MBT0729351.1 chain was the least hydrophilic (GRAVY = −0.150).

### 3.4 Structural properties of insect-associated bacterial phytase

For each source of phytase-producing bacteria—such as *B. horsfieldi* larvae, *H. illucens* larvae, *D. melanogaster*, and *A. mellifera* gut—one phytase enzyme was selected for three-dimensional structure prediction. The selected phytase met the following criteria: presence of a signal peptide predicted by both SignalP 6.0 and Phobius, isoelectric point (pI) greater than 5.5, instability index below 40, and a relatively high aliphatic index. SWISS-MODEL, based on homology, is faster and better suited for template-based modeling when reliable templates are available; otherwise, AlphaFold is used for protein modeling. AlphaFold uses deep learning for high-accuracy predictions, though it is computationally intensive (Waterhouse *et al.* 2018). Accordingly, homology-based three-dimensional models of AEQ29498.1, UOB87035.1, MBS0894709.1, and MBI0020902.1 were generated using SWISS-MODEL. The crystal structure of *Klebsiella* sp. ASR1 phytase (SMTL ID: 2wnh.1) was found to be the most suitable experimentally determined template for AEQ29498.1, UOB87035.1, MBS0894709.1, and MBI0020902.1.

In the Swiss model, the evaluation of model quality follows this order: (i) GMQE (Global Model Quality Estimate), (ii) QMEANDisCo global score, (iii) similarity, (iv) QMEAN Z-score, and (v) QSQE. The GMQE and QMEANDisCo global scores provide an overall assessment of model quality, ranging from 0 to 1, with higher values indicating better expected quality. A QMEAN Z-score close to 0.0 indicates a “native-like” model, while scores below −4.0 generally suggest poor model quality. A higher QSQE score, typically above 0.7, indicates a more reliable prediction of the quaternary structure (Waterhouse *et al.* 2018). Models of the full-length phytases showed lower quality compared to their mature phytase chain counterparts (Table 3). Among the models of mature phytases, the model of mature MBS0894709.1 displayed the highest QMEANDisCo Global score (0.76 ± 0.05), indicating the highest model quality.

**Table 3.** The model quality of mature phytase chains of insect-associated bacteria.

Phytase model	Length status	GMQE score	QMEANDisCo global score	Similarity (%)	QMEAN score	QSQE score
AEQ29498.1	Full length	0.72	0.74 ± 0.05	40.41	−1.32	0.49
	Mature	0.77	0.75 ± 0.5	40.41	−1.60	0.00
UOB87035.1	Full length	0.72	0.74 ± 0.05	37.96	−0.52	0.50
	Mature	0.76	0.75 ± 0.5	37.96	−0.87	0.52
MBS0894709.1	Full length	0.69	0.73 ± 0.05	42.43	−1.09	0.00
	Mature	0.77	0.76 ± 0.5	42.70	−0.52	0.51
MBI0020902.1	Full length	0.61	0.70 ± 0.05	34.65	−2.34	0.38
	Mature	0.66	0.70 ± 0.5	34.65	−2.71	0.40

**Figure 3.**
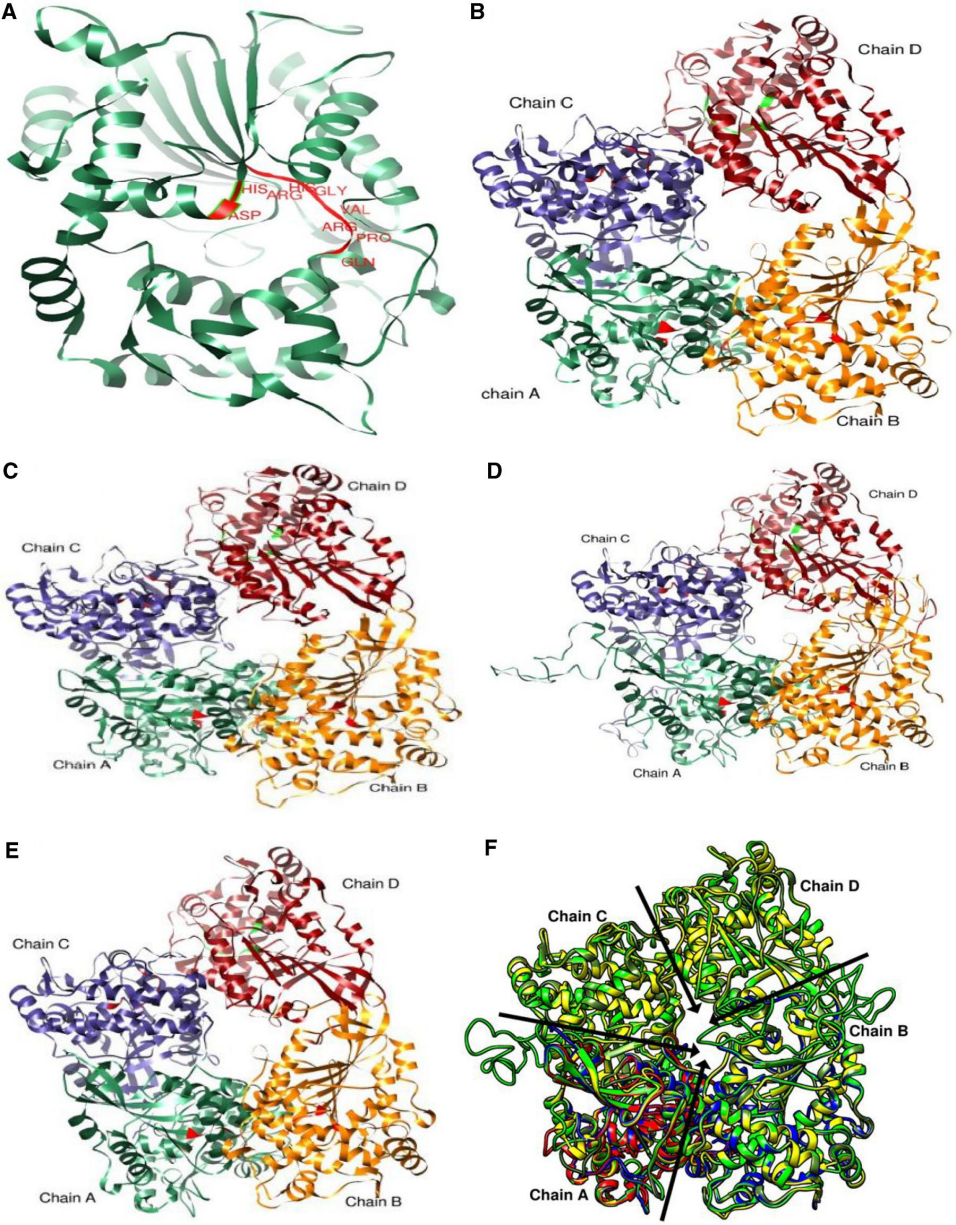
The three-dimensional structure of the matured phytase chains of insect-associated bacteria. (A) Monomeric structure of AEQ29498.1, featuring the catalytically active motif of histidine acid phosphatase (HAP). The homo-tetrameric structures of UOB87035.1 (B), MBS0894709.1 (C), and MBI0020902.1 (D) are displayed, with the catalytically active motif in each chain highlighted in red or green. (E) Quaternary structure (Chain A and B) of the template (SMTL ID: 2wnh.1) after the removal of Na^+^, Mg^2+^, and glycerol. (F) Superposition of models of AEQ29498.1, UOB87035.1, MBS0894709.1, MBI0020902.1 and 2wnh.1. AEQ29498.1 is red, UOB87035.1 is yellow, MBS0894709.1 is dark olive green, MBI0020902.1 is green and 2wnh.1 is blue. The models were visualized and modified using UCSF Chimera.

The best homology models of the mature phytase chains are shown in Fig. 3, while those of the full-length phytases are presented in Supplementary Fig. 1, available as supplementary data at *Bioinformatics Advances* online. The predicted tertiary structure revealed two distinct domains: the α/β domain and the α domain. The mature AEQ29498.1 chain was predicted to be a monomer (QSQE = 0.00). The predicted quaternary structures of the mature UOB87035.1, MBI0020902.1, and MBS0894709.1 were found to be homo-tetramers (Powers and Powers 2003), although the accuracy of these predicted homo-tetrameric assemblies was not strongly supported, as indicated by QSQE scores below 0.7 (Bertoni *et al.* 2017).

### 3.5 Families and functional properties of insect-associated bacterial phytase

Signal scanning of the full-length phytase sequences using InterPro classified all 27 sequences into Clade-2 (IPR00560) of the histidine phosphatase superfamily and the histidine acid phosphatase family (IPR050645). The catalytically active histidine acid phosphatase (HAP) family is characterized by two conserved motifs: the 'RHGxRxP'motif located in the N-terminal region and the “HD” motif in the C-terminal region (Kumar *et al.* 2012; Scott *et al.* 2024). The catalytic domain of HAP enzymes functions optimally under acidic pH conditions, a defining feature of this enzyme family (Rigden 2008). Within the RHGxRxP motif, a conserved histidine residue serves as a nucleophile during catalysis. The catalytic mechanism for phosphate group removal involves initially transferring the phosphate to the histidine residue, creating a phospho-histidine covalent intermediate that is subsequently hydrolyzed to release free phosphate (Nezhad *et al.* 2020). One example of HAP enzymes is phytases, which break down phytate, releasing phosphate.

**Figure 4.**
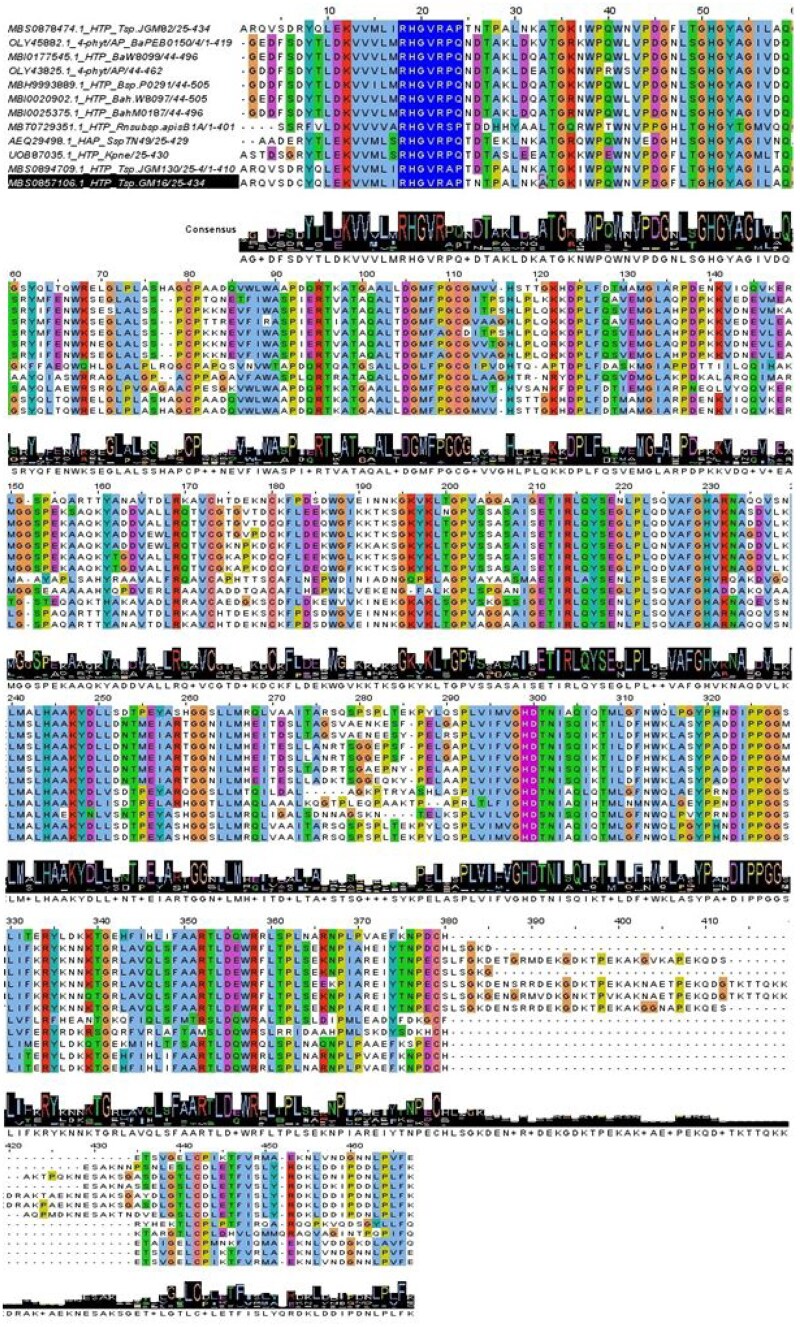
Multiple sequence alignment of the matured phytase chains of insect-associated bacteria. The blue color highlights the RHGxRxQ/P motif, while the violet color represents the HD motif. The MSA was performed using MUSCLE, and visualized and annotated using JALVIEW (Waterhouse *et al.* 2009).

In this study, 12 phytases with the same signal peptide predicted by SignalP 6.0 and Phobius were aligned using MUSCLE to analyze the conserved catalytic motifs. These mature phytases of insect-associated bacteria were found to contain the consensus sequences: “RHGVRP/AQ/P” at the N-terminal and “HD” at the C-terminal (Fig. 4). Similar consensus sequences were also observed in the full-length phytases (Supplementary Fig. 2, available as supplementary data at *Bioinformatics Advances* online). Eight mature phytases and 23 full-length phytases exhibited a P→Q substitution in the catalytic motif. This substitution in the highly conserved RHGxRxP of a HAP is likely functionally significant. In RHGxRxP motif, P typically plays a structural role, helping to properly position adjacent residues for catalysis. It contributes rigidity and restricts backbone flexibility, which is crucial for maintaining the shape of the active site. In contrast, Q is chemically very different: it is polar and capable of hydrogen bonding, whereas P is nonpolar and rigid. Thus, replacing P with Q would introduce a flexible, polar, and bulkier side chain, potentially disrupt the geometry and rigidity of the motif, and misaligning the catalytic histidine (H) or other residues critical for phosphate binding and hydrolysis. This change may affect local folding, substrate binding, or transition-state stabilization in the active site (Alberts *et al.* 2002).

**Figure 5.**
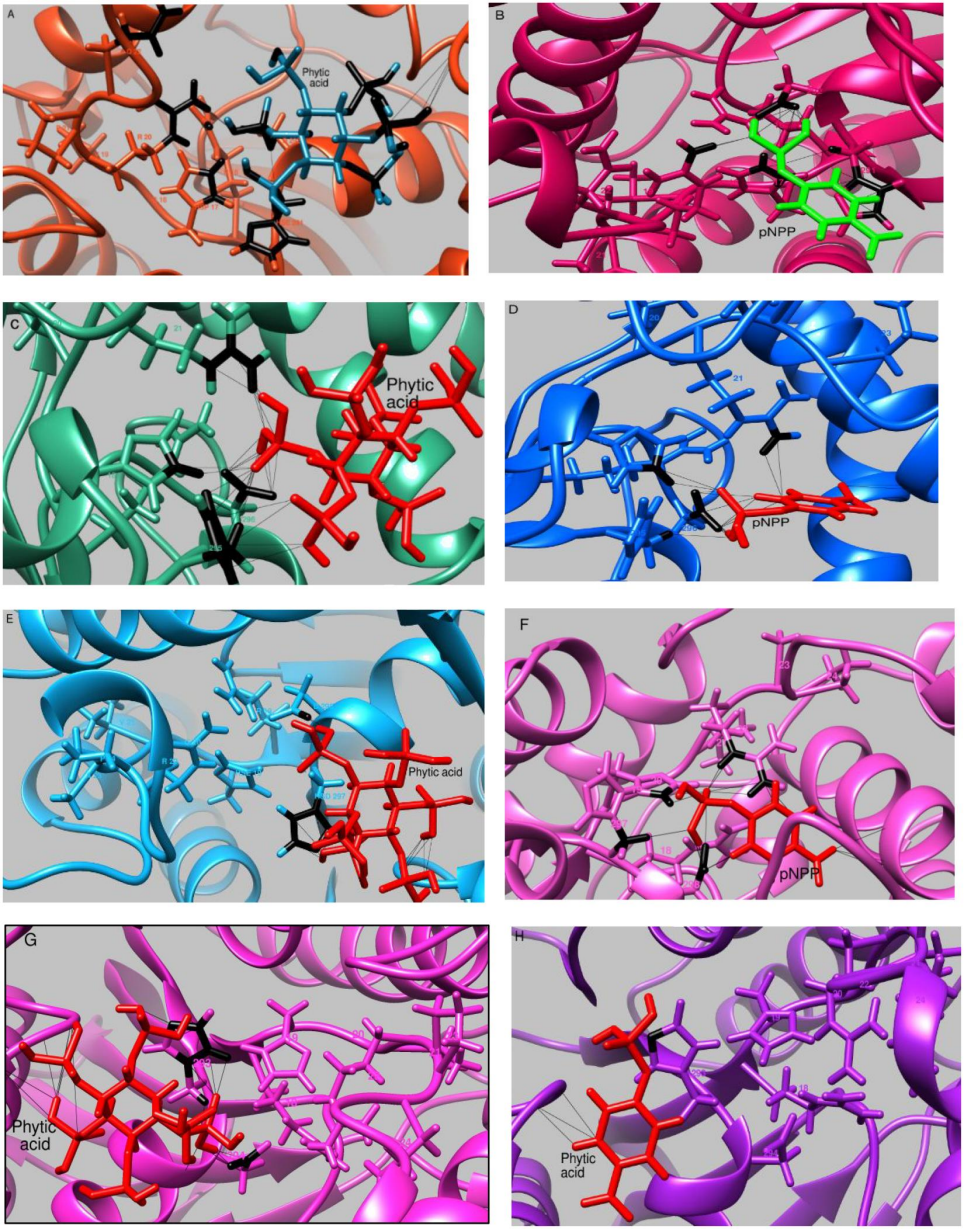
Molecular docking of substrates with insect-gut associated bacterial phytases. The molecular docking of phytic acid and *para*-nitrophenyl phosphate with AEQ29498.1 (A and B), with UOB87035.1 (C and D), with MBS0894709.1 (E and F) and MBI0020902.1 (G and H), respectively. The model was visualized and edited using UCSF Chimera.

Substrate affinity and specificity were investigated through molecular docking, where high-resolution crystal structures are preferred; however, when unavailable, homology models are used, their reliability depends on the sequence identity between the target and template (Bugnon *et al.* 2024). Additionally, docking mature proteins—which represent the biologically active form—is generally more accurate than using full-length proteins, as the latter may contain nonfunctional regions that introduce structural noise and reduce docking precision (Owji *et al.* 2018) Based on the results in Table 3, the mature models of AEQ29498.1, UOB87035.1, MBS0894709.1 and MBI0020902.1, which exhibited over 30% sequence similarity with the template, were docked with phytic acid and *para*-nitrophenyl phosphate, as shown in Table 4 and detailed in Fig. 5.

**Table 4.** The best poses exist at active site of insect-gut associated bacterial phytases.^a^

Model	Ligand	AC score	SP-DG score	Interacting residues
AEQ29498.1	PA^b^	−778.53	−7.54	6328 contacts including **ARG16**(1), **HSP17**(46), **ARG20**(312), **GLN22**(308), **HSE291**(297), **ASP292**(389)
	pNPP	−97.45	−5.80	**HSP17**(3), **ARG20**(2), LEU89(7), ARG91(14), GLN123(3), **HSE291**(7), **ASP292**(10), THR293(4)
UOB87035.1	PA	−779.94	−8.33	**HSE18** (2), **ARG21**(5), **ASN24**(20), ASP25(13), LYS28(10), ARG92(8), LYS193(2), LYS195(4), LYS244(4), LEU248(2), ASP249(4), **HSD**295(9), **ASP296**(8), THR297(3)
	pNPP	−100.89	−6.27	**HSE18**(2), **ARG21**(2), ASN24(5), ASP25(3), ARG92(7), SER204(7), GLU208(2), HSD241(2), LYS244(1), **HSD295**(1), **ASP296**(10), THR297(2)
MBS0894709.1	PA	−782.17	−8.41	PHE126(3), ASP127(6), THR128(3), MET129(3), LYS194(8), LEU249(3), TYR255(4), **HSD297**(12), **ASP298**(2), THR299(18), ASN300(1)
	pNPP	97.10	−5.93	**HSE19**(4), **ARG22**(12), ARG95(4), ALA205(4), ALA206(4), GLU209(6), HSD242(12), LYS245(20), TYR246(6), **HSD297**(4), **ASP298**(16), THR299(2)
MBI0020902.1	PA	−782.58	−8.45	ASP93(5), ARG95(7), PHE122(4), PHE126(4), LEU249(3), ASP127(17), THR128(4), ILE129(8), GLU130(1), LYS194(3), TYR255(4), **HSD293**(4), **ASP294**(3), SER295(7), ASN296(1), GLN299(1)
	pNPP	−99.76	−6.04	ASP93(5), ARG95(2), PHE126(1), ASP127(8), ILE129(1), TYR255(2), **HSD293**(1), SER295(7)

a Bold residues are part of catalytic motif; the numbers in the bracket show number of interactions.

b Abbreviations: PA = phytic acid; pNPP = 4-*para*-nitrophenyl phosphate.

The attractive cavity (AC) scores, which indicate the binding affinity between phytic acid and the phytase enzymes, were found to be −778.53, −779.94, −782.17, and −782.58 arbitrary units for AEQ29498.1, UOB87035.1, MBS0894709.1, and MBI0020902.1, respectively. These negative values reflect strong interactions between phytic acid and each phytase. Additionally, the binding of phytic acid to these phytases resulted in higher SwissParam-derived docking scores (SP-DG) compared to the binding of *para*-nitrophenyl phosphate, suggesting stronger and more favorable interactions with phytic acid (Table 4). In Swiss dock, AC and SP-DG represent free energy values. The AC score accounts for all energy contributions and is the preferred score for ranking poses of the same ligand with the same target. In contrast, the SP-DG score provides an approximate binding free energy of a ligand to a target. It is useful for comparing different target–ligand combinations but is a fast approximation and should not be used to rank poses of a single ligand with one target (Röhrig *et al.* 2023, Bugnon *et al.* 2024).

Among the phytases analyzed, MBI0020902.1—harboring the P24/Q24 mutation in the catalytic motif of histidine acid phosphatases—showed the lowest SP-DG value of −8.45 when interacting with phytic acid, indicating the highest binding affinity. Interestingly, MBS0894709.1, which retains the native catalytic motif of histidine acid phosphatases, exhibited the second-lowest SP-DG value. This result does not support a clear advantage of the P24/Q24 mutation in enhancing/reducing substrate affinity.

In the docked structures, the catalytic motifs were identified as follows: AEQ29498.1, 16-RHGVRPQ-22, and 291-HD-292; UOB87035.1, 18-RHGVRPQ-24, and 293-HD-294; MBS0894709.1, 18-RHGVRPQ-24, and 297-HD-298; and MBI0020902.1, 18-RHGVRPQ-24, and 293-HD-294. The histidine residues within these motifs were observed in different protonation states (HSP, HSE, and HSD) after docking. In the HSP (doubly protonated) state, both nitrogen atoms in the imidazole ring (Nε2 and Nδ1) carry a hydrogen atom, giving the residue a net positive charge (+1). In the HSE (singly protonated on Nε2) state, the hydrogen atom is located on the Nε2, resulting in a neutral charge. In the HSD (singly protonated on Nδ1) state, the hydrogen atom resides on Nδ1, which is also neutral. Functionally, protonated histidine can act as a general acid, neutral histidine can act as a nucleophile, deprotonated histidine can act as a general base during enzymatic catalysis (Kim *et al.* 2013).

## 4 Discussion

Phytase plays a crucial role in improving phosphorus bioavailability from plant-based feed ingredients, which is particularly important in monogastric animal nutrition such as poultry (Dersjant-Li *et al.* 2015, Urgessa *et al.* 2024). While soils, compost, and fermented foods have traditionally been the main sources of phytase discovery (Urgessa *et al.* 2024), the present study highlights insect gut microbiota as an underexplored but promising source of phytase-producing bacteria, given the phytate-rich nature of many phytophagous insect diets (Huerta-García and Álvarez-Cervantes 2024).

Due to the limited experimental and *in silico* investigations of insect-associated phytase-producing microbes, this study employed homology-based screening to identify potential phytase enzymes from bacteria inhabiting insect guts. The analysis addressed three key questions: (i) which insects harbor phytase-producing bacteria, (ii) which bacterial groups are involved, and (iii) what are the sequence, structural, and functional characteristics of the phytases they encode. Using the recombinant HAP reported by Zhang *et al.* (2011) as a query, we identified the gut microbiota of *Hermetia illucens* larvae, *Drosophila melanogaster*, and *Apis mellifera* as promising sources of phytase-producing bacteria (Table 1). These findings suggest that, beyond established habitats such as soil, animal digestive tracts, fermented foods, compost, manure, aquatic environments, and plant residues (Urgessa *et al.* 2024), insect guts represent valuable reservoirs of phytase-producing microbes. This broadens the ecological diversity of phytase sources and supports their potential applications in nutrition, environmental management, and agriculture (Dersjant-Li *et al.* 2015).

Although the phytate-rich diets of these insects imply that they may harbor diverse gut microbes capable of producing phytases, current knowledge remains limited. For instance, the black soldier fly (*H. illucens*) likely hosts substantial proportions of phytase-producing bacteria that support larval growth on phytate-rich substrates such as rotting plant matter and animal manure (Callegari *et al.* 2020). Similarly, while *D. melanogaster* larvae feed on decaying plant material, there are no reports of native phytase activity in their gut microbiota (Jelena *et al.* 2025), with phytase only demonstrated in genetically modified individuals (Retief *et al.* 2024). In the case of the Western honey bee (*A. mellifera*), which consumes phytic acid-rich nectar and pollen, studies on gut phytase activity are lacking, with most research focused instead on carbohydrate-active enzymes (Zheng *et al.* 2019). Likewise, in the longhorn beetle *Batocera horsfieldi*, only one phytase-producing bacterium (*Serratia* sp. TN49) has been reported (Zhang *et al.* 2011), underscoring major gaps in understanding phytase occurrence in insect-associated microbiota.

In this study, homology-based analysis revealed phytase-producing *Tatumella*, *Klebsiella*, *Bartonella*, and *Rosenbergiella* species inhabiting insect guts and mouthparts (Table 1). While *Klebsiella* and *Serratia* are relatively well-studied and known for feed and environmental applications (Escobin-Mopera *et al.* 2012, Pramanik et al. 2018b, Urgessa *et al.* 2024), the identification of *Tatumella*, *Rosenbergiella*, and *Bartonella* as represents novel and largely unexplored phytase sources.

Four phytases were selected for detailed computational analysis: AEQ29498.1 from *Serratia* sp. TN49 (isolated from the gut of *B. horsfieldi* larvae), UOB87035.1 from *K. pneumoniae* (found in the gut of *H. illucens* larvae), MBS0894709.1 from *Tatumella* sp. JGM130 (associated with the gut of *D. melanogaster*), and MBI0020902.1 from *B. apihabitans* W8097 (isolated from the gut of *A. mellifera*). Homology analysis identified annotation of UOB87035.1, MBS0894709.1, and MBI0020902.1 as “histidine-type phosphatases,” while AEQ29498.1 was specifically identified as a “histidine acid phytase” (Table 1). Histidine-type phosphatases form a subclass of acid phosphatases characterized by a conserved catalytic mechanism involving two motifs—RHGxRxP and HD (Figs. 4 and 5)—that are also characteristic of HAPs (Rigden, 2008, Nezhad *et al.* 2020). These enzymes hydrolyze phytic acid under acidic conditions, releasing inorganic phosphate (Kumar *et al.* 2012).

*In silico* protein sequence analysis further confirmed the presence of signal peptides in AEQ29498.1, UOB87035.1, MBS0894709.1, and MBI0020902.1 (Supplementary Table 2, available as supplementary data at *Bioinformatics Advances* online), indicating their potential for secretion. Additionally, the predicted biochemical characteristics of these enzymes—including acid stability, thermostability, and high solubility—make them well-suited to the poultry upper gastrointestinal tract (Table 2). Hydrolysis of phytate in the crop, proventriculus, and gizzard ensures early phosphorus release, enabling efficient absorption in the small intestine (Nezhad *et al.* 2020). Beyond phosphorus, early phytate hydrolysis also liberate essential minerals such as calcium, zinc, iron, and magnesium, forming which would otherwise be trapped in insoluble phytate complexes (Woyengo and Nyachoti 2013).

Moreover, phytate hydrolysis reduces its antinutritional effects, such as protein and enzyme binding, which can impair nutrient digestibility (Dersjant-Li *et al.* 2015). By breaking down phytate before chyme enters the small intestine, these phytases improve overall nutrient utilization. Environmentally, effective phytate hydrolysis also lowers phosphorus excretion in manure, reducing agricultural phosphorus pollution and promoting sustainable poultry production (Urgessa *et al.* 2024).

Molecular docking confirmed that all four insect-gut-associated phytases exhibited stronger predicted binding affinities toward phytic acid than toward 4-*para*-nitrophenyl phosphate (Table 4). Among them, MBI0020902.1 displayed the most favorable docking score (−8.45), followed closely by MBS0894709.1, indicating high substrate specificity. Conserved catalytic motifs—RHGxRxP (e.g. positions 16–22 or 18–24) and the HD dyad (e.g. 291–292 or 293–294)—were consistently identified across AEQ29498.1, UOB87035.1, MBS0894709.1, and MBI0020902.1. These motifs are hallmarks features of the histidine acid phosphatase family, which includes the most widely used phytases in feed applications (Chen *et al.* 2015). Their conservation confirms these presence of functional catalytic cores capable of efficient phytate recognition, binding, and hydrolysis under poultry gut conditions.

In the context of monogastric animal nutrition, phytate is the main phosphorus storage form in plant-based feed ingredients but is largely indigestible by these animals due to their limited endogenous phytase production (Woyengo and Nyachoti 2013, Kumar *et al.* 2019). The conservation catalytic motifs and favorable docking profiles across the four insect-gut-derived phytases demonstrate their functional potential and support their application as feed additives to enhance phosphorus bioavailability, improve nutrient absorption, and reduce environmental phosphorus excretion.

## 5 Conclusion

Among the 27 insect-associated phytases, four enzymes—AEQ29498.1 from *Serratia* sp. TN49, UOB87035.1 from *Klebsiella pneumoniae*, MBS0894709.1 from *Tatumella* sp. JGM130, and MBI0020902.1 from *Bartonella apihabitans* W8097—exhibited promising biochemical, structural, and functional characteristics. These phytases have potential as feed additives for monogastric animals, especially poultry, by enhancing nutrient absorption, reducing the need for inorganic phosphate supplementation, and improving feed efficiency. Importantly, with the growing the use of black soldier fly larvae (BSFL) in poultry diets, the presence of phytase-producing *K. pneumoniae* in the BSFL gut could further increase the nutritional value of BSFL-based feeds by lowering phytic acid content and enhancing mineral bioavailability. Overall, phytases derived from insect gut represent a valuable and underexplored resource for improving phosphorus utilization in animal nutrition. Future research should prioritize experimental validation to confirm these *in silico* predictions.

## Supplementary Material

vbaf256_Supplementary_Data

## Data Availability

Data are contained within the article and Supplementary Material.
