# Supplementary material for: In silico analysis of insect-associated bacterial phytases reveals optimal biochemical properties and function in poultry gut condition
Source: Bioinform Adv. 2025 Oct 15;5(1):vbaf256. doi: 10.1093/bioadv/vbaf256 (PMC12596144; doi:10.1093/bioadv/vbaf256)
Supplement: vbaf256_Supplementary_Data [file vbaf256_supplementary_data.zip › Supplementary Figure 1.doc]

Fig.S1. The 3D structure of the full-length phytases of insect-associated bacteria. A is the model of AEQ29498.1, B is the model of UOB87035.1, C is the model of MBS0894709.1 and D is the model MBI0020902.1. The 3D structure is composed of α-helices (cyan), extended-strand (Green) and random coils (White).
