# Supplementary material for: In silico analysis of insect-associated bacterial phytases reveals optimal biochemical properties and function in poultry gut condition
Source: Bioinform Adv. 2025 Oct 15;5(1):vbaf256. doi: 10.1093/bioadv/vbaf256 (PMC12596144; doi:10.1093/bioadv/vbaf256)
Supplement: vbaf256_Supplementary_Data [file vbaf256_supplementary_data.zip › Supplementary Figure 2.doc]

Fig. S2. Multiple sequence alignment (MUSCLE) of the 27 full-length phytases of insect-associated bacteria. Clustal colored output of Jalview indicate the RHGXRXQ (violet heptapeptide), and HD (blue dipeptide) motifs.
