# Supplementary material for: In silico analysis of insect-associated bacterial phytases reveals optimal biochemical properties and function in poultry gut condition
Source: Bioinform Adv. 2025 Oct 15;5(1):vbaf256. doi: 10.1093/bioadv/vbaf256 (PMC12596144; doi:10.1093/bioadv/vbaf256)
Supplement: vbaf256_Supplementary_Data [file vbaf256_supplementary_data.zip › Supplementary Table 1.doc]

**Supplementary Table 1**: Percent identiy matrix of the 27 phytase from insect-associated bacteria

|  | **1** | **2** | **3** | **4** | **5** | **6** | **7** | **8** | **9** | **10** | **11** | **12** | **13** | **14** | **15** | **16** | **17** | **18** | **19** | **20** | **21** | **22** | **23** | **24** | **25** | **26** | **27** |
| --- | --- | --- | --- | --- | --- | --- | --- | --- | --- | --- | --- | --- | --- | --- | --- | --- | --- | --- | --- | --- | --- | --- | --- | --- | --- | --- | --- |
| **1** | **100** |  |  |  |  |  |  |  |  |  |  |  |  |  |  |  |  |  |  |  |  |  |  |  |  |  |  |
| **2** | **90.5** | **100** |  |  |  |  |  |  |  |  |  |  |  |  |  |  |  |  |  |  |  |  |  |  |  |  |  |
| **3** | **92.48** | **93.86** | **100** |  |  |  |  |  |  |  |  |  |  |  |  |  |  |  |  |  |  |  |  |  |  |  |  |
| **4** | **92.34** | **92.74** | **92.34** | **100** |  |  |  |  |  |  |  |  |  |  |  |  |  |  |  |  |  |  |  |  |  |  |  |
| **5** | **91.73** | **91.53** | **93.55** | **92.34** | **100** |  |  |  |  |  |  |  |  |  |  |  |  |  |  |  |  |  |  |  |  |  |  |
| **6** | **91.53** | **91.94** | **92.34** | **91.94** | **93.55** | **100** |  |  |  |  |  |  |  |  |  |  |  |  |  |  |  |  |  |  |  |  |  |
| **7** | **89.72** | **90.93** | **92.14** | **92.74** | **93.15** | **93.95** | **100** |  |  |  |  |  |  |  |  |  |  |  |  |  |  |  |  |  |  |  |  |
| **8** | **88.71** | **89.11** | **90.32** | **90.73** | **91.13** | **91.73** | **93.95** | **100** |  |  |  |  |  |  |  |  |  |  |  |  |  |  |  |  |  |  |  |
| **9** | **89.31** | **91.33** | **91.73** | **93.55** | **91.13** | **92.94** | **94.76** | **94.76** | **100** |  |  |  |  |  |  |  |  |  |  |  |  |  |  |  |  |  |  |
| **10** | **86.9** | **89.11** | **89.52** | **88.71** | **89.31** | **89.72** | **89.92** | **88.71** | **89.52** | **100** |  |  |  |  |  |  |  |  |  |  |  |  |  |  |  |  |  |
| **11** | **89.72** | **88.91** | **87.9** | **89.92** | **90.32** | **89.92** | **89.11** | **88.71** | **89.52** | **93.15** | **100** |  |  |  |  |  |  |  |  |  |  |  |  |  |  |  |  |
| **12** | **88.34** | **88.34** | **89.78** | **89.98** | **88.75** | **87.73** | **90.18** | **90.18** | **90.39** | **86.3** | **85.07** | **100** |  |  |  |  |  |  |  |  |  |  |  |  |  |  |  |
| **13** | **88.31** | **88.96** | **89.18** | **87.66** | **88.53** | **89.18** | **88.74** | **87.23** | **88.96** | **90.26** | **89.18** | **86.36** | **100** |  |  |  |  |  |  |  |  |  |  |  |  |  |  |
| **14** | **89.18** | **89.83** | **89.83** | **89.18** | **90.26** | **88.53** | **88.53** | **86.8** | **89.39** | **88.31** | **88.31** | **87.66** | **91.77** | **100** |  |  |  |  |  |  |  |  |  |  |  |  |  |
| **15** | **88.53** | **89.61** | **89.61** | **88.31** | **88.31** | **88.74** | **89.83** | **88.1** | **90.04** | **89.61** | **87.88** | **88.1** | **90.69** | **91.56** | **100** |  |  |  |  |  |  |  |  |  |  |  |  |
| **16** | **87.66** | **89.61** | **90.04** | **87.45** | **88.74** | **89.61** | **88.96** | **88.96** | **90.04** | **90.69** | **89.39** | **88.31** | **91.99** | **91.77** | **93.07** | **100** |  |  |  |  |  |  |  |  |  |  |  |
| **17** | **86.58** | **88.53** | **89.39** | **86.58** | **88.1** | **88.31** | **88.1** | **87.88** | **89.18** | **90.04** | **88.31** | **87.66** | **91.99** | **93.29** | **92.42** | **98.05** | **100** |  |  |  |  |  |  |  |  |  |  |
| **18** | **87.01** | **88.96** | **89.83** | **87.01** | **88.53** | **88.74** | **88.53** | **88.31** | **89.61** | **90.48** | **88.74** | **88.1** | **92.42** | **92.64** | **93.29** | **98.92** | **99.13** | **100** |  |  |  |  |  |  |  |  |  |
| **19** | **87.23** | **87.23** | **88.1** | **88.96** | **86.15** | **87.45** | **90.04** | **89.61** | **90.69** | **87.01** | **85.5** | **89.18** | **89.39** | **88.74** | **90.48** | **89.61** | **89.18** | **89.39** | **100** |  |  |  |  |  |  |  |  |
| **20** | **84.48** | **85.89** | **85.89** | **87.1** | **83.87** | **84.48** | **87.9** | **87.1** | **88.31** | **84.68** | **83.27** | **88.34** | **90.26** | **91.34** | **91.34** | **90.48** | **90.91** | **90.26** | **91.56** | **100** |  |  |  |  |  |  |  |
| **21** | **84.25** | **85.48** | **85.69** | **86.91** | **84.46** | **84.66** | **87.93** | **87.12** | **88.34** | **84.87** | **83.03** | **90.12** | **89.18** | **89.61** | **90.91** | **89.39** | **89.83** | **89.18** | **90.48** | **93.87** | **100** |  |  |  |  |  |  |
| **22** | **45.05** | **44.58** | **45.75** | **44.81** | **44.81** | **45.05** | **45.05** | **45.28** | **44.81** | **43.87** | **44.58** | **45.05** | **45.05** | **44.1** | **44.58** | **45.28** | **45.28** | **45.28** | **45.28** | **44.34** | **44.1** | **100** |  |  |  |  |  |
| **23** | **48.59** | **46.95** | **47.18** | **48.36** | **47.18** | **47.42** | **47.31** | **47.89** | **47.89** | **47.89** | **47.89** | **47.89** | **48.12** | **47.18** | **46.71** | **47.18** | **46.95** | **46.95** | **47.42** | **47.89** | **47.89** | **48.56** | **100** |  |  |  |  |
| **24** | **48.71** | **48.71** | **49.41** | **48.95** | **49.18** | **48.95** | **49.65** | **48.24** | **48.48** | **48.24** | **48.95** | **48.48** | **49.65** | **48.71** | **48.01** | **48.71** | **48.48** | **48.48** | **48.01** | **48.71** | **49.18** | **47.49** | **52.49** | **100** |  |  |  |
| **25** | **49.07** | **49.53** | **50** | **49.77** | **48.84** | **49.53** | **50** | **49.77** | **50** | **49.77** | **49.53** | **49.3** | **51.16** | **49.3** | **47.67** | **49.07** | **48.84** | **48.84** | **49.77** | **49.77** | **49.53** | **49.88** | **52.47** | **70.47** | **100** |  |  |
| **26** | **48.6** | **49.07** | **49.53** | **49.3** | **48.37** | **49.07** | **49.53** | **49.3** | **49.77** | **49.3** | **49.07** | **48.84** | **50.7** | **48.84** | **47.21** | **48.6** | **48.37** | **48.37** | **49.3** | **49.3** | **49.07** | **49.17** | **52.24** | **70** | **99.08** | **100** |  |
| **27** | **48.6** | **49.07** | **49.53** | **49.3** | **48.37** | **49.07** | **49.53** | **49.3** | **49.77** | **49.3** | **49.07** | **48.84** | **50.7** | **48.84** | **47.21** | **48.6** | **48.37** | **48.37** | **49.3** | **49.3** | **49.07** | **49.41** | **52.47** | **70.23** | **99.31** | **99.77** | **100** |

Legend: 1 = histidine-type phosphatase (MBI0020902.1) of *Bartonella apihabitans* W8097; **2 =** histidine-type phosphatase of *Bartonella* sp. P0291(MBH9993889.1); **3 =** 4-phytase / acid phosphatase (AQT45424.10) of *Bartonella apihabitans* BBC0244; 4 = histidine-type phosphatase (MBI0168642.1) of *Bartonella* sp. W8167; 5 = histidine-type phosphatase (MBI0166446.1) of *Bartonella apihabitans* M0280; 6 = Bartonella apis B10834G6 (MBH9982862.1) of Bartonella apis B10834G6; 7 = 4-phytase/Alkaline Phosphatase (AQT43185.1) of *Bartonella apihabitans* BBC0178; 8 = histidine-type phosphatase (MBI0177545.1) of *Bartonella apis* W8099; 9= histidine-type phosphatase (MBI0162225.1) of *Bartonella* sp. M0283; 10 = histidine-type phosphatase (MBI0002091.1) of *Bartonella* sp. W8122; 11 = histidine-type phosphatase (MBI0025375.1) of *Bartonella apihabitans* M0187; 12 = histidine-type phosphatase (MBI0141245.1) of *Bartonella apis* W8099; 13 = histidine-type phosphatase (MBI0004047.1) of *Bartonella choladocola* B10834H15; 14 = 4-phytase/Alkaline Phosphatase (OLY43825.1) of *Bartonella* sp. M0283; 15 = 4-phytase/Alkaline Phosphatase (OLY47475.1) of *Bartonella apis* PEB0122; 16 = histidine-type phosphatase (MCT6861532.1) of *Bartonella apis* MRS1-bin.1; 17 = histidine-type phosphatase (MCT6887820.1) of *Bartonella apis* GUT-bin.12; 18 = histidine-type phosphatase (MCT6825384.1) of *Bartonella apis* MRS2-bin.4;19 = 4-phytase/Alkaline Phosphatase (OLY45882.1) of *Bartonella apis* PEB0150; 20 = histidine-type phosphatase (MBH9976047.1) of histidine-type phosphatase of Bartonella choladocola B10834H15; 21 = histidine-type phosphatase (AQT47836.1) of Bartonella choladocola BBC0122; 22 = histidine-type phosphatase (MBT0729351.1) of *Rosenbergiella nectarea* subsp. apis B1A; 23 = histidine acid phytase (AEQ29498.1) of *Serratia* sp. TN49; 24 = histidine-type phosphatase (UOB87035.1) of *Klebsiella pneumonia*; 25 = histidine-type phosphatase (MBS0894709.1) of Tatumella sp. JGM130; 26 = histidine-type phosphatase (MBS0857106.1) of Tatumella sp. JGM16; 27 = histidine-type phosphatase (MBS0878474.1) of Tatumella sp. JGM82
