# Supplementary material for: In silico analysis of insect-associated bacterial phytases reveals optimal biochemical properties and function in poultry gut condition
Source: Bioinform Adv. 2025 Oct 15;5(1):vbaf256. doi: 10.1093/bioadv/vbaf256 (PMC12596144; doi:10.1093/bioadv/vbaf256)
Supplement: vbaf256_Supplementary_Data [file vbaf256_supplementary_data.zip › Supplementary Table 2.doc]

**Supplementary Table 2.** **The predicted Sec/SPI of phytases from insect-associated bacteria**

| **No** | **Phytase NCBI Accession no** | **Position** | **Peptide sequences(N-terminal→C-terminal)** | **Predictive tool** |
| --- | --- | --- | --- | --- |
| 1 | AEQ29498.1 | 1-25 | MYKPMLSAALLALSASLLAATPAHA | Both |
| 2 | UOB87035.1 | 1-24 | MKKLLTLNLLLASITFSLIPASHA | Both |
| 3 | MBS0894709.1 | 1-24 | MKPLLILNLLLASLSLILSPFSSA | Both |
| 4 | MBS0878474.1 | 1-24 | MKPLLILNLLLASLSLILSPFSSA | Both |
| 5 | MBS0857106.1 | 1-24 | MKPLLILNLLLASLSLILSPFSSA | Both |
| 6 | MBI0004047.1 | NP | - | SignalP 6.0 |
|  |  | 1-37 | MKKSTDMSITTIRFLWAKLYCLMLFFIVVLLLGLSIS | Phobius |
| 7 | MBI0020902.1 | 1-43 | MKNSTDMSIATIRFLRAKICRLMAVFIVVLLLSLSISKPEAVA | Both |
| 8 | OLY45882.1 | 1-43 | MKKSTDMSIATIRFLWPKIFRLMPLFIVVLLLSLSISKPEALA | Both |
| 9 | MBI0168642.1 | 1-43 | MKKSTDMSIATIRFLRAKICRLMAVFIVVLLLSLSISKPEAIA | SignalP 6.0 |
|  |  | 1-37 | MKKSTDMSIATIRFLRAKICRLMAVFIVVLLLSLSIS | Phobius |
| 10 | MCT6861532.1 | 1-43 | MKKSTDLSITTIRFLWRKIYCLMLFFIVVLLLSLSISKPEAIA | SignalP 6.0 |
|  |  | 1-37 | MKKSTDLSITTIRFLWRKIYCLMLFFIVVLLLSLSIS | Phobius |
| 11 | MBI0002091.1 | NP | - | SignalP 6.0 |
|  |  | 1-37 | MKNSTDMSIATIRFLRAKIYCLMLFFIVVLLLSLSIS | Phobius |
| 12 | MBI0141245.1 | 1-43 | MKNSTDMSIATIRFLRVQICRLMAVFIVVLLLSLSISKPEAIA | SignalP 6.0 |
|  |  | 1-37 | MKNSTDMSIATIRFLRVQICRLMAVFIVVLLLSLSIS | Phobius |
| 13 | MBT0729351.1 | 1-26 | MPRSLIKNALIAWWVGLLSYVPFSDA | Both |
| 14 | MCT6887820.1 | NP |  | SignalP 6.0 |
|  |  | 1-37 | MKKSTDLSITTIRFLWRKIYCLMLFFIVVLLLGLSIS | Phobius |
| 15 | OLY43825.1 | 1-43 | MKKSTEMSITTIRFLWPKICRLMAVFIVVLLLGLSISKPEAIA | Both |
| 16 | MCT6825384.1 | NP |  | SignalP 6.0 |
|  |  | 1-37 | MKKSTDLSITTIRFLWRKIYCLMLFFIVVLLLGLSIS | Phobius |
| 17 | OLY47475.1 | 1-43 | MKKSTDMSMTTIRFLWAKICRLMAVFIVVLLTNFSISKPEAIA | SignalP 6.0 |
|  |  | 1-37 | MKKSTDMSMTTIRFLWAKICRLMAVFIVVLLTNFSIS | Phobius |
| 18 | MBI0162225.1 | 1-43 | MKKSTDMSIATIRFLRAKICRLMAVFIVVLLLSLSISKPEAIA | SignalP 6.0 |
|  |  | 1-37 | MKKSTDMSIATIRFLRAKICRLMAVFIVVLLLSLSIS | Phobius |
| 19 | MBH9976047.1 | NP | - | SignalP 6.0 |
|  |  | 1-37 | MKKSTDMSMTTIRFLWPKLYCLMAVFIVVLLLSLSIS | Phobius |
| 20 | MBI0177545.1 | 1-43 | MKNSTDMSIATIRFVCRKIFRLKLVFIFVLLLSLSISKPEAIA | Both |
| 21 | AQT45424.1 | 1-43 | MKKSTDMSIATIRFLLVQICRLMAVFIVVLLLSLSISKPEAIA | SignalP 6.0 |
|  |  | NP |  | Phobius |
| 22 | MBI0025375.1 | 1-43 | MKKSTDISIATILFLRAKIYCLKLVFIFVLLLSLSISKPEAIA | Both |
| 23 | AQT43185.1 | NP |  | Both |
| 24 | MBI0166446.1 | 1-43 | MKKSTDMSIATIRFLRVQICRLMAVFIVVLLLSLSISKPEAIA | SignalP 6.0 |
|  |  | 1-37 | MKKSTDMSIATIRFLRVQICRLMAVFIVVLLLSLSIS | Phobius |
| 25 | MBH9982862.1 | 1-43 | MKNSTDMSIATIRFLRAKICRLMAVFIVVLLLSLSISKPEAIA | SignalP 6.0 |
|  |  | 1-37 | MKNSTDMSIATIRFLRAKICRLMAVFIVVLLLSLSIS | Phobius |
| 26 | AQT47836.1 | NP |  | SignalP 6.0 |
|  |  | 1-37 | MKKSTDMSMTTIRFLWAKLYCLMAVFIVVLLLSLSLS | Phobius |
| 27 | MBH9993889.1 | 1-43 | MKKSTDMSIATIRFLRVQICRLMAVFIVVLLLSLSISKPEAIA | Both |

NP stands for Not Predicted. Red font indicates the differing signal peptide residues predicted for the same phytase by SignalP 6.0 and Phobius.
