# Supplementary material for: In silico analysis of insect-associated bacterial phytases reveals optimal biochemical properties and function in poultry gut condition
Source: Bioinform Adv. 2025 Oct 15;5(1):vbaf256. doi: 10.1093/bioadv/vbaf256 (PMC12596144; doi:10.1093/bioadv/vbaf256)
Supplement: vbaf256_Supplementary_Data [file vbaf256_supplementary_data.zip › Supplemetary Table 3.doc]

Supplementary Table 3: Physicochemical properties of full-length phytases from insect-associated bacteria

| No | NCBI Accession | MW | pI | Cys | Ab | | II | AI | GRAVY |
| --- | --- | --- | --- | --- | --- | --- | --- | --- | --- |
|  |  |  |  |  | S-S | SH | **<**40 |  |  |
| 1 | AEQ29498.1 | 46.638 | 9.05 | 6(1.4%) | 1.185 | 1.177 | 37.11 | 81.89 | -0.244 |
| 2 | UOB87035.1 | 47.018 | 6.84 | 6(1.4%) | 1.229 | 1.221 | 36.88 | 82.19 | -0.322 |
| 3 | MBS0894709.1 | 47.439 | 8.43 | 6(1.4%) | 1.249 | 1.242 | 38.27 | 89.52 | -0.204 |
| 4 | MBS0878474.1 | 47.483 | 8.63 | 6(1.4%) | 1.248 | 1.240 | 37.54 | 88.62 | -0.215 |
| 5 | MBS0857106.1 | 47.430 | 8.38 | 7(1.6%) | 1.250 | 1.242 | 37.89 | 88.62 | -0.199 |
| 6 | MBI0004047.1 | 51.296 | 6.34 | 7(1.5%) | 1.379 | 1.372 | 30.29 | 85.52 | -0.267 |
| 7 | MBI0020902.1 | 55.572 | 7.56 | 7(1.4%) | 1.021 | 1.015 | 29.25 | 83.09 | -0.383 |
| 8 | OLY45882.1 | 51.223 | 5.87 | 6(1.3%) | 1.186 | 1.179 | 33.60 | 86.99 | -0.264 |
| 9 | MBI0168642.1 | 54.944 | 6.25 | 7(1.4%) | 1.006 | 0.999 | 33.01 | 81.83 | -0.400 |
| 10 | MCT6861532.1 | 51.463 | 6.39 | 7(1.5%) | 1.346 | 1.338 | 31.03 | 86.15 | -0.303 |
| 11 | MBI0002091.1 | 55.256 | 7.11 | 7(1.4%) | 1.127 | 1.120 | 36.40 | 82.24 | -0.410 |
| 12 | MBI0141245.1 | 54.133 | 6.14 | 7(1.4%) | 1.014 | 1.021 | 33.94 | 83.03 | -0.363 |
| 13 | MBT0729351.1 | 47.079 | 7.27 | 6(1.4%) | 1.502 | 1.495 | 43.12 | 86.23 | -0.099 |
| 14 | MCT6887820.1 | 51.328 | 6.53 | 7(1.5%) | 1.242 | 1.235 | 31.93 | 85.30 | -0.304 |
| 15 | OLY43825.1 | 50.842 | 6.13 | 7(1.5%) | 1.195 | 1.188 | 35.23 | 86.80 | -0.236 |
| 16 | MCT6825384.1 | 51.391 | 6.81 | 7(1.5%) | 1.240 | 1.233 | 31.32 | 85.30 | -0.321 |
| 17 | OLY47475.1 | 51.262 | 5.92 | 7(1.5%) | 1.185 | 1.178 | 30.81 | 83.61 | -0.278 |
| 18 | MBI0162225.1 | 54.879 | 6.58 | 7(1.4%) | 1.034 | 1.027 | 34.39 | 83.41 | -0.371 |
| 19 | MBH9976047.1 | 55.004 | 5.77 | 7(1.4%) | 1.132 | 1.125 | 34.76 | 82.42 | -0.356 |
| 20 | MBI0177545.1 | 54.943 | 6.35 | 7(1.4%) | 1.033 | 1.026 | 34.52 | 82.42 | -0.364 |
| 21 | AQT45424.1 | 55.832 | 7.54 | 8(1.6%) | 1.019 | 1.010 | 33.75 | 79.62 | -0.446 |
| 22 | MBI0025375.1 | 54.722 | 6.20 | 7(1.4%) | 1.064 | 1.058 | 32.64 | 86.17 | -0.349 |
| 23 | AQT43185.1 | 57.708 | 6.01 | 8(1.5%) | 1.986 | 0.977 | 32.59 | 83.02 | -0.359 |
| 24 | MBI0166446.1 | 54.829 | 6.54 | 7(1.4%) | 1.035 | 1.028 | 31.24 | 83.02 | -0.365 |
| 25 | MBH9982862.1 | 54.970 | 8.44 | 7(1.4%) | 1.032 | 1.026 | 32.30 | 83.02 | -0.428 |
| 26 | AQT47836.1 | 54.192 | 6.15 | 7(1.4%) | 1.149 | 1.142 | 34.62 | 82.62 | -0.345 |
| 27 | MBH9993889.1 | 56.118 | 6.78 | 7(1.4%) | 1.109 | 1.103 | 34.33 | 80.59 | -0.458 |

MW is molecular weight in kDa; pI isoelectric point in pH, Cys is Cysteine amino acid composition of the matured phytase sequence. ab is predicted light absorbance at 280 nm in water by 1 g/L of matured phytase assuming all pairs of Cys residues form Cystine (S-S), or are reduced (SH). II is instability index, AI is aliphatic index, and GRAVY is Grand average of hydropathicity.
